# Poor sleep quality and anxiety in patients with diabetes mellitus and its association with glycemic control: A cross-sectional study

**DOI:** 10.1371/journal.pone.0361467

**Published:** 2026-11-11

**Authors:** Wissal Baghdad, Amal Satté, Otmane El Harrati, Mohamed Yassine El Brouzi, Zakaria Bernoussi, Amine Raggabi, Ihsane Hmamouchi, Naoual El Omri, Fadoua Mekouar, Abdelhalem Mesfioui, Youssef Sqalli Houssaini, Aboubaker El Hessni

**Affiliations:** 1 Department of Biology, Faculty of Sciences, Laboratory of Biology and Health, Neuroscience, Neuro-Immunology and Behavior Unit, Ibn Tofail University, Kenitra, Morocco; 2 Department of Neurophysiology, Mohammed V Military Instruction Hospital, Rabat, Morocco; 3 Department of Internal Medicine, Mohamed V Military Instruction Hospital, Rabat, Morocco; 4 Department of Biology, Faculty of Sciences, Physiology and Physiopathology Research Team, Genomics of Human Pathology Research Center, Mohammed V University, Rabat, Morocco; 5 Department of Musculoskeletal Diseases, Hôpital UIR, Salé, Morocco; 6 Health Sciences Research Centre (CReSS), International University of Rabat (UIR), Rabat, Morocco; Johns Hopkins: Johns Hopkins University, UNITED STATES OF AMERICA

## Abstract

**Background/objective:**

Sleep is crucial for maintaining physical and mental health, and poor sleep quality is associated with an increased risk of metabolic, cognitive, and cardiovascular diseases. This study aimed to assess sleep quality and factors associated with poor sleep quality, such as mental disorders, especially anxiety, in adult patients with type 2 diabetes in Morocco.

**Patients and methods:**

A cross-sectional study was conducted on patients with type 2 diabetes, using random selection, aged 39–85, in the clinical neurophysiology and internal medicine department of the Mohammed V Military Instruction Hospital, between 30/09/2024 and 30/12/2024. The questionnaire comprised demographic data, the Pittsburgh Sleep Quality Index (PSQI), and the Generalized Anxiety Disorder Scale (GAD-7). Descriptive tests, independent two-sample t-tests, chi-square tests, and logistic regressions were performed using Jamovi version 2.6.2.

**Results:**

Out of the 120 participants, the mean age of our patients was 65.4 ± 9.32 years, of whom 66.67% were women. The mean PSQI score was 9.18 ± 3.73, and the mean GAD-7 score was 8.0 ± 6.11. Poor sleep quality was reported in 84.2% of participants, while a binary logistic regression analysis revealed that poor sleep quality was not significantly associated with high HbA1c, but rather was highly significant with age and GAD-7.

**Conclusion:**

The study reveals that poor sleep quality is common among type 2 diabetic patients in Morocco, and is strongly associated with generalized anxiety disorders. These findings underscore the crucial role of psychological factors in diabetes management. They highlight the need for a comprehensive and integrated approach that takes into account not only biomedical aspects but also the psychological and behavioral dimensions of patients.

## 1. Introduction

Sleep is a natural state of mental and physical recess vital for preserving overall health and well-being [1]. Although there is a lack of understanding about the precise purpose of sleep, it is widely recognized as essential for cognitive, emotional, and physiological processes [2]. Poor sleep quality can significantly impact both physical and mental health, resulting in daytime fatigue, irritability, and concentration difficulties [3]. Recent studies have reported that the long-term consequences of poor sleep include an increased risk of obesity, diabetes mellitus (DM) (especially type 2 diabetes (T2D)), poor glycemic control, and cardiovascular diseases [4–6].

A link between poor sleep quality and DM has been reported in previous studies [7–12]. DM, one of the most prevalent chronic diseases, is a group of metabolic diseases characterized by persistently elevated blood glucose levels, insufficient insulin production, or insulin resistance [13,14]. Worldwide, diabetes affected 285 million people aged between 20 and 79 in 2010 (6.4% of this age group), and is expected to affect almost 439 million people (7.7%) in 2030.

Most people with diabetes have T2D, which accounts for 85–95% of all cases [15]. Rapid social and cultural changes, such as aging populations, urbanization, dietary changes, decreased physical activity, and unhealthy behaviors, contribute to their prevalence. Leading to a lower quality of life and reduced survival for those affected [16,17].

Most sleep disorders have been well defined, but poor sleep quality is still a nonspecific concept that does not correspond to any of the criteria presented by the International Classification of Sleep Disorders (ICSD), despite its epidemiological and clinical relevance. Poor sleep quality is a frequent complaint in the general population [18,19] and has been associated with comorbid metabolic, cognitive, and cardiovascular conditions [20].

In the general population, sleep disorders are frequently observed throughout the world [21]. In Morocco, a country in the International Diabetes Federation (IDF's) MENA region, the prevalence of diabetes is considerable. The 10^th^ edition of the Diabetes Atlas (2021) and the Moroccan League against Diabetes report that out of an adult population (20–79 years) of 23,992,800, there are 2,327,700 adults with diabetes, a prevalence of 9.7% [22]. Diabetes can disrupt sleep quality by altering fasting plasma glucose levels, especially during nocturnal hypoglycemia, and symptoms, including frequent urination and thirst, which cause nighttime awakening. In addition, diabetes complications such as neuropathy and restless legs syndrome can lead to sleep disturbances [7,23]. Sleep quality, which is often poor among diabetic patients, can be improved by optimizing glycemic control and symptom management [24]. On the other hand, sleep disorders can have a negative impact on diabetes control [15].

The World Health Organization (WHO) has set a target of a 25% reduction in avoidable mortality from noncommunicable diseases, including diabetes, by 2025 [25], with the management of poor sleep quality as a key intervention.

Poor sleep quality complicates the clinical management of diabetes, necessitating an understanding of its extent and risk factors to prevent, identify, and treat it effectively. Sleep disorders are common in T2D patients, particularly physiological imbalances and comorbid sleep disorders, leading to poor sleep quality and adverse health outcomes [23].

Poor sleep quality in diabetic patients can reduce their capacity to work. It also affects cognitive performance and can increase the risk of stroke, anxiety, and depression [25]. Sleep disorders are common in patients with T2D, as are symptoms of depression and anxiety. The combined effect of sleep disorders, depressive symptoms, and anxiety disorders has been shown to increase the risk of poor glycemic control [5]. Previous studies in T2D patients provided substantial evidence and reported a reciprocal correlation between poor sleep quality and glycemic control.

However, despite the recognized impact of sleep disorders, particularly insomnia which has a prevalence of 30.2% in Morocco [26], poor sleep, which is one of the forms of these disorders, has not received particular attention in previous research regarding its incidence and impact on glycemic control among the Moroccan diabetic population. In Morocco, sleep medicine has only been practiced for 23 years; although the number of sleep centers is increasing due to growing patient demand, the need for epidemiological data is evident. A cross-sectional study on the impact of poor sleep quality on glycemic control in Morocco is therefore necessary.

To our knowledge, no local study conducted among the Moroccan population has yet examined the impact of poor sleep quality in patients with T2D and its association with psychiatric factors using the Pittsburgh Sleep Quality Index (PSQI) and the Generalized Anxiety Disorder (GAD-7) scale. Our study is the first to assess the association of poor sleep quality with glycemic control and anxiety in the Moroccan diabetic population.

According to this study [26], which revealed that the prevalence of sleep disorders in Morocco shows marked regional disparities, with Fez-Meknes having the highest rate (23.76%), followed by Beni Mellal-Khenifra (11.88%) and Marrakech-Safi (11.28%). In contrast, Rabat-Sale-Kenitra had the lowest rate (0.29%), which may reflect better access to care.

We therefore conducted a regional cross-sectional study in the Rabat region to assess the prevalence of poor sleep quality and associated factors, such as mental health disorders, among Moroccan patients with T2D at the Mohammed V Military Instruction Hospital (HMIMV) in Rabat, Morocco. We also assessed the impact of sleep quality on glycemic control and psychological factors in a Moroccan adult population with T2D. We sought to determine whether there were correlations between subjective sleep quality and glycemic control, on the one hand, and psychological factors, on the other.

## 2. Materials and methods

### 2.1. Participants and procedures

The present cross-sectional study was designed to identify the factors that affect sleep quality in 120 patients with T2D admitted to the internal medicine department of the HMIMV, in Rabat, Morocco, between 30/09/2024 and 30/12/2024.

#### 2.1.1. Inclusion criteria.

Consisted of any type 2 diabetic patient (age ≥ 18 years), diagnosed >1 year ago, under regular treatment for diabetes disease, scheduled for consultation or hospitalization in the internal medicine department at HMIMV, Rabat, during the study period, and who had previously undergone biological analysis and agreed to participate in this study (Fig 1).

**Fig 1:**
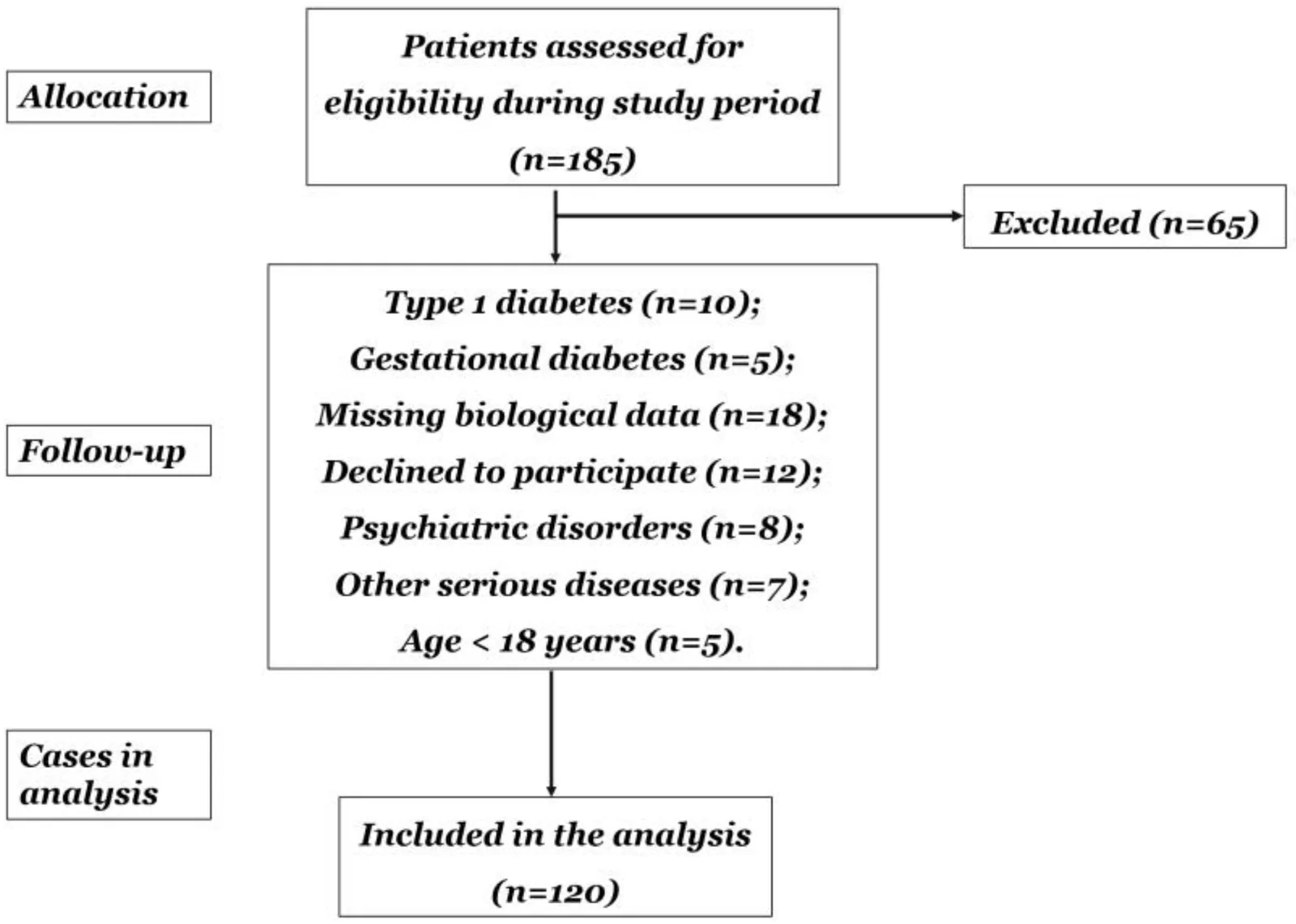
Flowchart of participant selection and inclusion in the cross-sectional study.

#### 2.1.2. Exclusion criteria.

Any patient with type 1 diabetes mellitus, gestational diabetes, a patient who had not previously undergone biological testing and who came for consultation during the study period, who lacked interest in participating in the study. Diabetic patients with any psychiatric or neurological disorders or other serious diseases were also excluded because these conditions significantly affect sleep quality [27], and to minimize potential confounding effects associated with established clinical conditions (Fig 1).

### 2.2. Clinical measurements and biochemical analysis

We assessed body mass index (BMI) derived from: weight (mass in kilograms)/height2 (in meters) and laboratory tests, including; (1) Glycemic control: glycosylated hemoglobin A1c (HbA1c) level, fasting plasma glucose levels, and (2) lipid profile levels: low-density lipoprotein cholesterol (LDL), high-density lipoprotein cholesterol (HDL), triglycerides (TG), and total cholesterol (TC).

### 2.3. Data collection

Data collection was conducted over a three-month period, from 30/09/2024–30/12/2024, during the internship. Data were collected using a structured questionnaire comprising sociodemographic and clinical variables, the Pittsburgh Sleep Quality Index (PSQI), and the Generalized Anxiety Disorder-7 (GAD-7) scale. The questionnaire was administered through structured interviews to ensure standardized data collection. It was initially prepared in French and translated into Moroccan Arabic to facilitate participants’ understanding.

Clinical and laboratory data, including biochemical parameters, were obtained from the most recent laboratory assessment performed before the interview. For hospitalized patients, these data were retrieved from their medical records. Missing information was obtained from medical records when available. Data completeness was checked throughout data collection, data entry, and analysis.

In addition to the PSQI and GAD-7, the questionnaire included an additional question regarding perceived sleep need. Participants were asked, “How many hours of sleep do you think you need?” This question was administered independently of the PSQI and assessed participants’ perceived sleep requirement rather than the sleep-duration component of the PSQI. No specific recall period was specified because participants were asked to report their perceived need for sleep. For descriptive purposes, responses were classified into three categories: short perceived sleep need (<7 h/night), moderate perceived sleep need (7–9 h/night), and long perceived sleep need (>9 h/night). This additional measure was not used in the calculation of the PSQI score.

#### 2.3.1. General information questionnaire: Sociodemographic data sheet.

The basic demographic information of participants included: Age, gender, educational level, marital status, residence, lifestyle (smoking, coffee consumption, and physical exercise activity), employment status, and smartphone/interactive media use. Height and weight were measured during the consultation on the first day of admission to the service to calculate BMI.

In addition, data on family history of diabetes, duration of diabetes mellitus in years, number of hospitalizations due to diabetes, the mode of diabetes diagnosis was assessed based on the circumstances leading to the identification of diabetes. Participants were categorized into three groups: diagnosis following diabetes-related complications, diagnosis prompted by diabetes-related symptoms, and incidental diagnosis during routine screening or medical evaluation unrelated to diabetes symptoms. This information was collected through structured interviews and verified using available medical records. Therapeutic regimen, medical history, and diabetes-related comorbidities were also recorded, including hypertension, dyslipidemia, obesity, hyperuricemia, autoimmune diseases (including autoimmune thyroiditis “Hashimoto’s thyroiditis”, Graves ‘disease, systemic lupus erythematosus, and rheumatoid arthritis), and cardiovascular diseases. Biochemical parameters such as fasting plasma glucose, HbA1c (%), TC, HDL, LDL, and TG levels were recorded during the interview based on the most recent laboratory assessment performed before the interview. For hospitalized patients, these laboratory results were obtained from their clinical medical records. The questionnaire served as a data collection tool to recording the available clinical and biochemical information.

#### 2.3.2. Assessment of sleep quality.

The first measure used in the present study was the PSQI, an internationally validated questionnaire that evaluates subjective sleep quality and disturbances during the month preceding the survey [28]. The PSQI score is calculated from 19 items divided into seven subcategories of sleep quality, each rated on a 0–3 point scale (no difficulty to severe difficulty). The total score for each subcategory constitutes the PSQI total score, which varies on a scale from 0 to 21 points (from no difficulty to major difficulty). An overall PSQI score above 5 has been validated as having a high sensitivity of 89.6% and specificity of 86.5% for distinguishing good from poor sleepers in some populations [28], including people with T2D [29].

#### 2.3.3. Specific anxiety questionnaire.

The second scale in the present study was the GAD-7, a 7-item self-report tool used to screen participants for anxiety and the severity of GAD-7 over the last two weeks [30]. Every item was scored between 0 and 3 points. The overall scores can vary between 0 and 21. According to a recent meta-analysis, the threshold recommended by experts for estimating generalized anxiety is 8 to optimize sensitivity without compromising specificity.

### 2.4. Ethical considerations

The present study was carried out with respect to the dignity and ethics of the participants. Data collection was carried out in accordance with patient confidentiality. All procedures in this work involving patients complied with the ethics standards specified by the national and institutional research committee. All participants received information on the project's nature, context, and objectives and signed an informed consent form. We obtained all the official authorizations required, including the approval of the ethics committee, which referenced CERB 132−24, the hospital director, and the heads of the clinical neurophysiology and internal medicine departments. Confidentiality and privacy were guaranteed to all participants. We have respected the Moroccan Arabic dialect [31,32].

### 2.5. Statistical analysis

Descriptive statistical analysis methods (median, mean, frequency, ratio, standard deviation, minimum, maximum) were used to evaluate the study data and were analyzed using Jamovi version 2.6.2. The data are expressed as the mean ± SD for continuous variables and as percentages for categorical variables. Mean ± SD and frequency (%) were used in descriptive analysis to describe patients’ sociodemographic and medical characteristics, sleep quality, and anxiety scores.

Sociodemographic, biochemical, and clinical variables were analyzed with a chi-square (χ^2^) test for comparisons of categories and a student’s t-test for continuous measures. The seven PSQI components were analyzed to identify the prevalence of good/bad sleepers. The Mann-Whitney U test was used for variables that were not normally distributed. All associations with a significance level were P ≤ 0.05.

We performed binary logistic regression to determine the significant predictors of poor sleep quality (coded 1). We included significant variables (i.e., age, HbA1C values, GAD-7 score) in the preliminary analysis of the model. The odds ratio (OR) and Pearson's correlation coefficient were used to identify sleep-related factors in the analysis. As the GAD-7 and HbA1c variables do not follow a normal distribution, we used Spearman's correlation coefficient (rho) to assess their relationships.

## 3. Results

### 3.1. Descriptive characteristics

The present study was carried out on 120 diabetic patients, referred to the Mohamed V Military Teaching Hospital, Rabat, Morocco, in 2024. The sociodemographic and clinical characteristics of the study participants are presented in (Table 1). Overall, 120 patients with T2D were included in the study, with a response rate of 100%. Their ages ranged from 39 to 85 years (65.4 ± 9.32 years), and more than half of the participants (66.67%) were women. Also, 75% were married. The average number of children was 4.04 ± 1.96. Forty-five percent were still working. A total of 1.7% were current smokers, and 71.7% did not engage in physical exercise.

**Table 1:** Characteristics of the study Participants at HMIMV. (N = 120).

Variables	N=120
	n (%)
**Sociodemographic**	
**Gender**	
**Man**	40 (33.3)
**Women**	80 (66.7)
**Age group (years)**	65.4 ± 9.32
**<65 years**	56 (46.7)
**> 65 years**	64 (53.5)
**Marital status**	
**Divorced**	2 (1.7)
**Married**	90 (75.0)
**Single**	1 (0.8)
**Widow**	27 (22.5)
**Educational status**	
**Illiterate**	60 (50.0)
**Primary School**	35 (29.2)
**Secondary School**	23 (19.2)
**Hight School**	2 (1.7)
**Occupation**	
**Unemployed +retired**	66 (55.0)
**Employed (part/full time)**	54 (45.0)
**Residence**	
**Urban**	118 (98.3)
**Rural**	2 (1.7)
**BMI (Kg/m^2^)**	
**Underweight**	1 (1.2)
**Normal BMI**	23 (26.7)
**Overweight**	33 (38.4)
**Obese**	29 (33.7)
**Life style behaviors**	
**Coffee consumption**	73 (60.8)
**Smoking history**	
**Current Smokers**	2 (1.7)
**Never**	99 (82.5)
**Weaned**	19 (15.8)
**Physical activity**	
**Inactive**	86 (71.7)
**Active**	34 (28.3)
**Sleep History**	
**History of sleep disturbance**	38 (31.7)
**Prior diagnoses of sleep disturbance**	15 (12.5)
**Perceived sleep need**	
**Short sleep**	40 (33.3)
**Moderate sleep**	66 (55)
**Long sleep**	14 (11.7)
**Trigger for sleep disorders**	
**Psychological causes**	32 (40.5)
**Physiological causes**	32 (40.5)
**Hygiene-related causes**	2 (2.5)
**Environmental causes**	13 (16.5)
**Mental status**	
**Presence of depression**	23 (19.2)
**Presence of stress disorders**	87 (72.5)
**Presence of memory problems**	86 (71.7)
**Presence of concentration problems**	75 (63.0)
**Presence of fatigue**	40 (33.3)
**GAD-7 Anxiety (Scores)**	6 [3,15]
**GAD-7 Anxiety (Scores)**	
**Minimal Anxiety**	51 (42.5)
**Mild Anxiety**	24 (20.0)
**Moderate Anxiety**	14 (11.7)
**Severe Anxiety**	31 (25.8)
**Environmental factor**	
**Exposure to light at night**	28 (23.3)
**Use of social networks during the night**	40 (33.9)
**Watching TV at night**	39 (32.8)
**Nocturnal eating habits**	
**Eat at night**	33 (27.5)
**Snack at night**	27 (22.5)

*Note(s): GAD-7 scores: 0–4 = **Minimal Anxiety**/ 5–9 = **Mild Anxiety**/10–14 = **Moderate Anxiety**/ > 15 = **Severe Anxiety**. Physical activity: **Active** (≥30 min/day and ≥ 3times/ week) / **Inactive** (<30min/day and <3times/week). **Perceived sleep need:** refers to the number of hours of sleep participants reported they believed they needed each night. **Short sleep** (SSD: < 7hours/night), **Moderate sleep** (MSD: 7–9 hours/night), **Long sleep** (LSD: > 9 hours/night) BMI (Kg/m^2^): **Underweight** (BMI < 18.5), **Normal BMI** (18.5 − 24.99), **Overweight** (25–29.9), or **Obese** (BMI ≥ 30). **“Never smoked”** refers to patients who do not smoke. “**Weaned”** refers to patients who have smoked 100 cigarettes or more in their lifetime, but who have not smoked in the past year. **“Night-time eating”** was defined as the consumption of a regular meal during the night, whereas **“night-time snacking”** referred to the consumption of smaller amounts of food or beverages (e.g., snacks) between meals during the night. **“BMI”** was available for 86 participants; 34 participants declined weight and/or measurements. Percentages are based on participants with available BMI data.

Regarding BMI, 33.7% of the patients were obese, while 26.7% were within the normal BMI range. The mean (± SD) BMI of the patients was 29.1 ± 5.75 Kg/m^2^. In terms of anxiety status, 42.5% of the 120 T2D patients had “Minimal” scores, while 11.7% had “Moderate” scores and 25.8% had “Severe” anxiety symptoms, based on the GAD-7 Anxiety Score. All the environmental factors, including exposure to light at night, night-time social networking/ interactive media, and TV watching, associated with nocturnal eating habits, can fragment and disrupt sleep quality (a major cause of difficulty sleeping / insomnia). The data revealed that 28 patients had frequent exposure to night light, 13 because of habits, 6 due to fear, and 1 due to stress.

### 3.2. Status of diabetes control, comorbidities

Nineteen participants (16%) received insulin alone, while oral antidiabetic drugs were the most commonly used treatment, reported in 63.9% of participants. Regarding the mode of diabetes diagnosis, 29.7% of participants were diagnosed following the occurrence of diabetes-related complications, 37.3% were diagnosed after experiencing diabetes-related symptoms, and 33.1% were diagnosed incidentally during routine screening or medical evaluation unrelated to diabetes symptoms.

The mean ± SD HbA1c was 8.02 ± 1.63%, and the mean ± SD duration of diabetes mellitus was 15.40 ± 9.61 years. With a standard deviation of 1.25 years, diabetic patients were hospitalized an average of 1.95 times, with a maximum of 6 times. Furthermore, seventy-three percent of the participants had poor glycemic control (HbA1c level >7%) (Table 2).

**Table 2:** Medical characteristics of Diabetes Control, Comorbidities among participants (N = 120).

Variables	N = 120
Duration of diabetes (Years)*	15.40 ± 9.61
Duration of diabetes (Years) ** < 5 years 5-10 years > 10 years	16 (13.3) 27 (22.5) 77 (64.2)
Number of hospitalization due to diabetes*	1.95 ± 1.25
Diabetic medication ** OHA (Oral drugs combination) Insulin Only (Injection therapy) OHA+ Insulin (Both) Diet	76 (63.9) 19 (16.0) 20 (16.8) 4 (3.4)
HbA1c (%) *	8.02 ± 1.63
HbA1c (%) ** Uncontrolled (≥7)	80 (66.66)
fasting plasma glucose level (g/l) *	1.52 ± 0.53
fasting plasma glucose level (g/l) ** Abnormal levels (≥1.10 g/l)	87 (83.7)
Lipid control	
HDL Level (g/l) *	0.49 ± 0.14
HDL Level (g/l) ** Abnormal levels (Man: < 0.4g/l) Abnormal levels (Woman: < 0.5g/l)	6 (46.2) 17 (44.7)
LDL Level (g/l) *	1.09 ± 0.36
LDL Level (g/l) ** Abnormal Levels (>1.5g/l)	7 (14.0)
TG Level (g/l) *	1.43 ± 0.61
TG Level (g/l) ** Abnormal Levels (>1.5g/l)	17 (32.7)
CT Level (g/l) *	1.81 ± 0.47
CT Level (g/l) ** Abnormal Levels (>2g/l)	18 (34.6)
Comorbidity	
HTA	81 (67.5)
Dyslipidemia	48 (40.0)
Obesity	55 (45.8)
Hyperuricemia	54 (45.0)
Auto-immune diseases	14 (11.7)
Cardiovascular diseases	40 (33.3)

Note (s): *: Mean± SD; **: N (%) Duration of diabetes: A period ≤ 10 years since first recognition is considered “short» duration, and a period > 10 years is considered “long duration.” Comorbidity: Presence of at least one or more medical problems in an individual along with Diabetes mellitus. The lipid profile thresholds have been established according to laboratory ROCHE reference values.

Among the comorbidities in the patients, hypertension (HTA) was the most common, with 67.5%, while obesity was the second highest at 45.8%, and 40% showed dyslipidemia. Cardiovascular disease was diagnosed in 40 participants (33.3%). A total of 83.7% had abnormal fasting plasma glucose levels, and 10% had at least one lipid level abnormality. These patients were classified as having poorly controlled diabetes.

### 3.3. Prevalence of poor sleep quality

The mean duration of sleep disturbances, as reported by all participants, was 4.07 ± 3.69 years. Based on the results represented in Table 3, the mean ±SD of the global PSQI score was 9.18 ± 3.73. Regarding subjective sleep quality, 14.2% of patients reported very poor subjective sleep quality, while 46.7% had fairly good sleep quality. Regarding sleep latency, 50.8% reported a latency of more than 60 min. In terms of sleep duration, 10.8% reported for less 5 hours per night, while 16.7% reported a sleep duration of 6–7 hours. Additionally, 30% of the patients had a sleep efficiency below 85%.

**Table 3:** Number and proportion of seven Components of PSQI Score among 120 Patients with T2D at HMIMV (N = 120).

Sleep Quality Index (PSQI)		T2D Total (N=120)
T-PSQI Mean ± SD		9.18±3.73
Component of PSQI	Category	
**Subjective sleep quality**	Very good (0)	10 (8.3)
	Fairly good (1)	56 (46.7)
	Fairly bad (2)	37 (30.8)
	Very bad (3)	17 (14.2)
**Sleep latency (min)**	<15min (0)	10 (8.3)
	16-30min (1)	13 (10.8)
	31-60min (2)	36 (30.0)
	>60min (3)	61 (50.8)
**Sleep duration (h)**	>7h (0)	59 (49.2)
	6-7h (1)	20 (16.7)
	5-6h (2)	28 (23.3)
	<5h (3)	13 (10.8)
**Habitual Sleep efficiency (%)**	>85% (0)	84 (70.0)
	75-84% (1)	21 (17.5)
	65-74% (2)	9 (7.5)
	<65% (3)	6 (5.0)
**Sleep disturbance**	Never (0)	3 (2.5)
	< 1-times/week (1)	59 (49.2)
	1-2 times/ week (2)	50 (41.7)
	Use 3-4 times/ week (3)	8 (6.7)
**Use of sleep medication**	Never (0)	105 (87.5)
	< 1-times/week (1)	1 (0.8)
	1-2 times/week (2)	1 (0.8)
	Use 3-4 times/week (3)	13 (10.8)
**Daytime dysfunction**	Never (0)	20 (16.7)
	< 1-times/week (1)	14 (11.7)
	1-2 times/week (2)	19 (15.8)
	Three times/week (3)	67 (55.8)

Additionally, 49.2% of the patients experienced severe sleep disturbances at least once a week, 10.8% used sleeping pills at least three to four times a week, and 55.8% exhibited daytime dysfunction at least three to four times a week. The total prevalence of poor sleep quality (global PSQI score > 5) in diabetic patients was 84.2% (101/120), with 26.7% (32/101) in men and 57.5% (69/101) in women

The most frequently reported sleep disturbance occurring three or more times per week in (Table 4) were ‘having to get up to go to the bathroom’ and ‘not being able to fall asleep in less than 30 minutes’, followed by ‘waking up in the middle of the night or early in the morning’ and ‘Feeling Hot’. Getting up to use the toilet is the most common complaint among T2D patients. Most of the participants reported having a partner who sleeps in the same room but not in the same bed.

**Table 4:** Excessive daytime sleepiness in poor and good sleepers with type 2 diabetes.

Sleep disturbances for three or more times a week	T2D Total (N = 120)	Good quality (N = 19)	Poor quality (N = 101)
Have to get up to use bathroom	93 (77.5)	4 (3.3)	89 (74.2)
Cannot get to sleep within 30 min	89 (74.2)	5 (4.2)	84 (70.0)
Wake up in the middle of night or early morning	56 (46.7)	3 (2.5)	53 (44.2)
Cough or snore loudly	22 (18.3)	4 (3.3)	18 (15.0)
Night mare	35 (29.2)	1 (0.8)	34 (28.3)
Pain	9 (7.5)	1 (0.8)	8 (6.7)
Feel hot	41 (34.2)	1 (0.8)	40 (33.3)
Cannot breathe comfortably	23 (19.2)	0 (0)	23 (19.2)
Feel Cold	27 (22.5)	0 (0)	27 (22.5)

**Notes:** *p < 0.05, **p < 0.01, ***p < 0.001.

### 3.4. Relationship between blood glucose control and PSQI score

The relationship between glycemic control and PSQI components was investigated (Table 5). Patients were classified into groups as having good glycemic control (HbA1c < 7%) or poor glycemic control (HbA1c ≥ 7%) according to their HbA1c. The mean global PSQI score for overall study population was 9.18 ± 3.73. Among all patients included in the study, only 40 (33.3%) were classified as having good glycemic control. No statistically significant differences were observed between the two groups for subjective sleep quality, sleep duration, habitual sleep efficiency, sleep disturbances, use of sleep medication, or global PSQI score (all P > 0.05). Daytime dysfunction was the only PSQI component that differed significantly between the two groups, with higher scores among participants with inadequate glycemic control (2.30 ± 1.10 vs 1.79 ± 1.15, P *=* 0.01).

**Table 5:** PSQI scores according to glycemic control in patients with T2D at HMIMV. (N = 120).

Variables	Good glycemic Control (N=40)	Uncontrolled diabetes (N=80)	Overall (N=120)	P-Value
**1. Subjective Sleep Quality**	1.48±0.78	1.49±0.84	1.51±0.84	**0.93**
**2. Sleep Latency**	2.17±1.07	2.24±0.93	2.23±0.95	**0.70**
**3. Sleep Duration**	1.00±1.07	0.91±1.09	0.95±1.08	**0.85**
**4. Habitual Sleep Efficiency**	0.62±1.01	0.38±0.73	0.47±0.84	**0.37**
**5. Sleep Disturbances**	1.62±0.62	1.51±0.69	1.52±0.66	**0.67**
**6. Use of Medication**	0.31±0.93	0.33±0.92	0.35±0.95	**0.86**
**7. Daytime Dysfunction**	1.79±1.15	2.30±1.10	2.11±1.16	**0.01**
**Global Sleep Quality (PSQI)**	9.00±4.15	9.20±3.66	9.18±3.73	**0.48**

**Notes:** *p < 0.05, **p < 0.01, ***p < 0.001.

### 3.5. Comparing general information between poor and good sleepers

A comparison of general information between poor and good sleepers was conducted (Table 6). Patients were classified as poor sleepers (PSQI >5) and good sleepers (PSQI ≤ 5) based on their overall PSQI score. The proportion of patients classified as poor sleepers (PSQI >5) was 84.2%. In addition, the duration of diabetes, BMI, age, lipid control, family history of diabetes, number of hospitalizations due to diabetes, and HbA1c were higher in poor sleepers than in good sleepers. The percentage of patients using insulin in the poor sleeper group was higher than that in the good sleeper group, without a significant statistical difference. Twenty percent of poor sleepers are frequently exposed to night light, while the rate of good sleepers who are exposed to night light is 3.3%. Poor sleepers watch more television at night than good sleepers do. Poor sleepers eat more than good sleepers do during the night and snack a lot. The study showed that GAD-7 scores and HDL-C levels differed significantly between participants with poor and good sleep quality (P < 0.01 and P < 0.05, respectively), while the remaining comparisons were not statistically significant.

**Table 6:** Characteristics of participants according to sleep quality (N = 120).

Item	Poor Sleeper (n=101)	Good Sleeper (n=19)	Overall (n=120)	P-Value
Categorical determinants*				
**Family history**	49 (40.8)	9 (7.5)	58 (48.3)	**0.92**
**Smoking**	2 (1.7)	0 (0.0)	2 (1.7)	**0.09**
**Use of Insulin**	14 (11.8)	5 (4.2)	19 (16.0)	**0.13**
**Physical activity**	28 (23.3)	6 (5.0)	34 (28.3)	**0.73**
**Coffee drink**	62 (51.7)	11 (9.2)	73 (60.8)	**0.77**
**Exposure to light at night**	24 (20.0)	4 (3.3)	28 (23.3)	**0.79**
**Use of social networks during the night**	35 (29.7)	5 (4.2)	40 (33.9)	**0.55**
**Watching TV at night**	35 (29.4)	4 (3.4)	39 (32.8)	**0.23**
**Eat at night**	29 (24.2)	4 (3.3)	33 (27.5)	**0.49**
**Snack at night**	23 (19.2)	4 (3.3)	27 (22.5)	**0.86**
Continuous determinants**				
**Age (year)**	65.8±8.91	63.3±11.3	65.4±9.32	**0.28**
**BMI (Kg/m^2^)**	29.2±5.92	28.5±4.89	29.1±5.75	**0.70**
**Duration of diabetes mellitus (years)**	15.5±9.07	15.0±12.4	15.40±9.61	**0.57**
**Number of hospitalization due to diabetes**	2.04±1.30	1.64±1.08	1.95±1.25	**0.29**
**fasting plasma glucose level (g/L)**	1.51±0.494	1.59±0.743	1.52±0.53	**0.55**
**TC (g/L)**	1.85±0.44	1.61±0.601	1.81±0.47	**0.19**
**TG (g/L)**	1.41±0.64	1.53±0.509	1.43±0.619	**0.60**
**LDL-C (g/L)**	1.12±0.34	0.93±0.44	1.09±0.36	**0.19**
**HDL-C (g/L)**	0.50±0.14	0.40±0.13	0.49±0.14	**0.04**
**Glycemic control HbA1C (%)**	8.07±1.64	7.79±1.59	8.02±1.63	**0.52**
**Duration of sleep disorders (years)**	4.19±3.98	3.46±1.66	4.07±3.69	**0.52**
**GAD-7 Score**	8.64±6.24	4.58±3.98	8.0±6.11	**0.007**

Note: *: N (%). **: Mean±SD.

### 3.6. Prevalence of anxiety

Table 7, shows the mean and SD of the GAD-7 questionnaire and its subscales. The mean (SD) overall GAD-7 score was 8.00 ± 6.11. In the GAD-7 questionnaire, the highest and lowest scores were related to feeling nervous, anxious, or on edge, and feeling afraid as if something awful might happen, with scales of 1.71 ± 1.22 and 0.36 ± 0.74, respectively. Good sleepers had an average GAD-7 score of 4.58 ± 3.98, while poor sleepers had an average GAD-7 score of 8.64 ± 6.24. Feeling nervous, anxious, or on edge was highly significant (P < 0.001) in poor sleepers, feeling unable to stop or control worry was significant (P < 0.05), and feeling too much worry about too many different things was significant (P < 0.05) in our patients.

**Table 7:** Generalized Anxiety Disorder (GAD-7) questionnaire with their sub-scales.

Component of GAD-7	Type 2 DM Total (N=120)	Total Good sleep quality (N=19)	Total Poor sleep quality (N=101)	P- Value
**Feeling nervous, anxious or on edge**	1.71±1.22	0.68±1.11	1.90±1.14	**<0.001**
**Not being able to stop or control worrying**	1.60±1.14	0.89±1.10	1.73±1.10	**0.003**
**Worrying too much about different things**	1.56±1.23	1.00±1.20	1.66±1.21	**0.03**
**Trouble relaxing**	0.97±1.07	0.78±0.97	1.01±1.09	**0.41**
**Being so restless that it is hard to sit still**	1.04±1.26	0.63±0.95	1.12±1.30	**0.16**
**Becoming easily annoyed or irritable**	0.75±1.11	0.42±0.83	0.81±1.15	**0.21**
**Feeling afraid as if something awful might happen**	0.36±0.74	0.15±0.50	0.40±0.77	**0.17**
**Total of GAD-7**	8.00±6.11	4.58±3.98	8.64±6.24	**0.01**

**Notes:** *p < 0.05, **p < 0.01, ***p < 0.001.

Table 8 presents the components of the GAD-7 score in 120 T2D patients at HMIMV. Among these patients, 52 (43.3%) were classified as anxious with a total GAD-7 score above 8, while 68 (56.7%) were classified as not anxious with a score below 8. Additionally, the results reported that regarding the different aspects of anxiety assessed, 47 patients (39.2%) felt nervous, anxious, or on edge almost every day. In addition, 37 patients (30.8%) reported not being able to stop or control their worries almost every day, and 42 patients (35.0%) worried excessively about various things almost every day. Difficulty relaxing was reported by 17 patients (14.2%) almost every day, while 28 patients (23.3%) experienced such agitation that they found it difficult to sit still most days. Furthermore, 16 patients (13.3%) felt easily irritated or irascible almost every day. Finally, only 2 patients (1.7%) experienced intense fear, as if something terrible was going to happen, almost every day. The present study suggests that a significant proportion of T2D patients at HMIMV present various levels of anxiety, with a notable number suffering from persistent nervousness, excessive worry, and difficulty relaxing.

**Table 8:** Number and Proportion of Seven Components of the GAD-7 Score among 120 Patients with T2D at HMIMV (N = 120).

Global Generalized Anxiety Disorder GAD-7 score		Type 2 DM (N=120)	Absence of generalized anxiety (<8) (N=68)	Generalized anxiety disorder (>8) (N=52)	P-Value
T-GAD-7 Mean ± SD		8.00±6.11	3.22±2.17	14.3±3.27	
Component of GAD-7	Category	N (%)	N (%)	N (%)	
**Feeling nervous, anxious, or on edge**	Not at all (0)	29 (24.2)	29 (24.2)	0 (0)	**<0 .001**
	Several days (1)	24 (20.0)	23 (19.2)	1 (0.8)	
	More than half the days (2)	20 (16.7)	8 (6.7)	12 (10.0)	
	Nearly every day (3)	47 (39.2)	8 (6.7)	39 (32.5)	
**Not being able to stop or control worrying**	Not at all (0)	26 (21.7)	26 (21.7)	0 (0)	**<0 .001**
	Several days (1)	33 (27.5)	32 (26.7)	1 (0.8)	
	More than half the days (2)	24 (20.0)	9 (7.5)	15 (12.5)	
	Nearly every day (3)	37 (30.8)	1 (0.8)	36 (30.0)	
**Worrying too much about different things**	Not at all (0)	33 (27.5)	33 (27.5)	0 (0)	**<0 .001**
	Several days (1)	29 (24.2)	28 (23.3)	1 (0.8)	
	More than half the days (2)	16 (13.3)	5 (4.2)	11 (9.2)	
	Nearly every day (3)	42 (35.0)	2 (1.7)	40 (33.3)	
**Trouble relaxing**	Not at all (0)	53 (44.2)	50 (41.7)	3 (2.5)	**<0 .001**
	Several days (1)	34 (28.3)	14 (11.7)	20 (16.7)	
	More than half the days (2)	16 (13.3)	4 (3.3)	12 (10.0)	
	Nearly every day (3)	17 (14.2)	0 (0)	17 (14.2)	
**Being so restless that it is hard to sit still**	Not at all (0)	64 (53.3)	56 (46.7)	8 (6.7)	**<0 .001**
	Several days (1)	15 (12.5)	8 (6.7)	7 (5.8)	
	More than half the days (2)	13 (10.8)	4 (3.3)	9 (7.5)	
	Nearly every day (3)	28 (23.3)	0 (0)	28 (23.3)	
**Becoming easily annoyed or irritable**	Not at all (0)	76 (63.3)	59 (49.2)	17 (14.2)	**<0 .001**
	Several days (1)	14 (11.7)	6 (5.0)	8 (6.7)	
	More than half the days (2)	14 (11.7)	3 (2.5)	11 (9.2)	
	Nearly every day (3)	16 (13.3)	0 (0)	16 (13.3)	
**Feeling afraid as if something awful might happen**	Not at all (0)	93 (77.5)	62 (51.7)	31 (25.8)	**<0 .001**
	Several days (1)	12 (10.0)	3 (2.5)	9 (7.5)	
	More than half the days (2)	13 (10.8)	3 (2.5)	10 (8.3)	
	Nearly every day (3)	2 (1.7)	0 (0)	2 (1.7)	

**Notes:** *p < 0.05, **p < 0.01, ***p < 0.001.

### 3.7. Comparison of general information between anxious and no anxious patients

When comparing participants with and without generalized anxiety disorder, a statistically significant difference in age was observed between the two groups. However, no statistically significant difference was observed for the other sociodemographic variables, or with the overall PSQI score, as shown in Table 9.

**Table 9:** Determinants of anxious and non-anxious Patients with T2D at HMIMV (N = 120).

Item	Generalized anxiety disorder (n=52)	Absence of generalized anxiety (n=68)	Overall (n=120)	P-Value
Categorical determinants*				
**Family history of diabetes mellitus**	25 (20.8)	33 (27.5)	58 (48.3)	**0.96**
**Smoking**	1 (0.8)	1 (0.8)	2 (1.7)	**0.81**
**Use of Insulin**	7 (5.9)	12 (10.1)	19 (16.0)	**0.56**
**Physical activity**	14 (11.7)	20 (16.7)	34 (28.3)	**0.76**
**Coffee drink**	32 (26.7)	41 (34.2)	73 (60.8)	**0.89**
**Exposure to light at night**	16 (13.3)	12 (10.0)	28 (23.3)	**0.09**
**Use of social networks during the night**	21 (17.8)	19 (16.1)	40 (33.9)	**0.14**
**Watching TV at night**	18 (15.1)	21 (17.6)	39 (32.8)	**0.61**
**Eat at night**	16 (13.3)	17 (14.2)	33 (27.5)	**0.48**
**Snack at night**	14 (11.7)	13 (10.8)	27 (22.5)	**0.31**
Continuous determinants **				
**Age (year)**	63.4±9.21	66.9±9.17	65.4± 9.32	**0.04**
**BMI (Kg/m^2^)**	29.4±5.90	28.8±5.67	29.1±5.75	**0.60**
**Duration of diabetes mellitus (years)**	15.2±8.66	15.6±10.3	15.40±9.61	**0.81**
**Number of hospitalizations due to diabetes**	2.0±1.36	1.91±1.18	1.95±1.25	**0.78**
**fasting plasma glucose level (g/L)**	1.50±0.48	1.53±0.57	1.52±0.53	**0.80**
**TC (g/L)**	1.92±0.46	1.72±0.45	1.81±0.47	**0.11**
**TG (g/L)**	1.37±0.62	1.48±0.62	1.43±0.61	**0.55**
**LDL-C (g/L)**	1.19±0.38	1.01±0.33	1.09±0.36	**0.08**
**HDL-C (g/L)**	0.51±0.18	0.47±0.10	0.49±0.14	**0.40**
**Glycemic control HbA1C (%)**	7.84±1.51	8.17±1.72	8.02±1.63	**0.29**
**Duration of sleep disorders (years)**	4.64±4.95	3.63±2.28	4.07±3.69	**0.24**
**PSQI Score**	9.60±3.52	8.85±3.87	9.18±3.73	**0.28**

Note: *: N (%). **: Mean±SD.

### 3.8. Correlations

The GAD-7 score was weakly and positively correlated with the global PSQI score (r = 0.150; P ≤ 0.05), while HbA1C was not correlated with the PSQI sleep quality score (r = 0.082, p = 0.399). Similarly, no significant correlation was observed between HbA1C and GAD-7 scores (r = −0.038, p = 0.695). the correlation results are presented in Table 10.

**Table 10:** Correlation Between Sleep Quality Index, HbA1c, and Anxiety Score Among Patients with T2D at HMIMV (N = 120).

	Last HbA1C	PSQI Total Score	GAD-7 Total score
**Last HbA1C**	-	0.082 α (p=0.399)	-0.038 β (p=0.695)
**PSQI Total score**	0.082 α (p=0.399)	-	0.150 β (p=0.042) *
**GAD-7 Total score**	-0.038 β (p=0.695)	0.150 β (p=0.042) *	-

**Notes: α: r Pearson and β: Rho Spearman.**

### 3.9. Predictors of poor sleep quality

Multivariable logistic regression analysis revealed that age and anxiety symptoms are significant predictors of poor sleep quality (Table 11). For each additional year of age, the estimated odds of poor sleep quality were 6% higher (adjusted OR=1.06; CI:1.00 to 1.14; P = 0.04), holding the other variables in the model constant. Similarly, each one-point increase in the GAD-7 score was associated with a 21% increase in the estimated odds of poor sleep quality (adjusted OR=1.21; 95% CI:1.06 to 1.39; P = 0.005). In contrast, HbA1c was not a significant predictor of poor sleep quality (adjusted OR= 1.13; 95% CI: 0.79 to 1.64; P = 0.48). Gender and night-time social media use were also not statistically significant predictors of poor sleep quality (adjusted OR= 0.61; 95% CI: 0.18 to 2.03; P = 0.42 and adjusted OR=1.06; 95% CI: 0.30 to 3.71; P = 0.92, respectively). In the multivariable logistic regression model, age and anxiety symptoms were independently associated with higher odds of poor sleep quality, after adjustment for glycemic control (P = 0.04 and P = 0.005, respectively).

**Table 11:** Multivariable logistic regression model of Poor Sleep Quality (PSQI>5) Among Patients with T2D at HMIMV (N = 120).

*Variables*	*Adjusted OR*	*95% CI*		*P-value*
		*Lower*	*Upper*	
***Age***	1.06	[1.00; 1.14]		0.04*
***Gender (Female= Ref)***	0.61	[0.18; 2.03]		0.42
***Global GAD-7 scores***	1.21	[1.06; 1.39]		0.005**
***HbA1C***	1.13	[0.79; 1.64]		0.48
***Night-time social media use (yes=ref)***	1.06	[0.30; 3.71]		0.92

**Notes:** Variable(s) entered as continuous variables and in step 1 are age, HbA1C, and Global GAD-7 scores. Female was used as the reference category for gender, and yes was used as the reference category for night-time media use. Adjusted ORs represent the change in the odds of poor sleep quality (PSQI>5) associated with a one-unit increase in each continuous predictors, holding the other variables in the model constant. **Notes:** *p<0.05, **p<0.01, ***p<0.001.

## 4. Discussion

Sleep quality is an integral part of health quality. Poor night sleep is associated with depression, anxiety, impaired social performance, chronic illness, and mortality [33]. In addition, poor sleep quality is associated with an increased risk of insulin resistance and complications in T2D patients. A bidirectional relationship exists between sleep and diabetes management. Improved sleep management can increase the chances of improved diabetes management, and vice versa [25]. Difficulty in initiating or maintaining sleep increases the risk of developing T2D twofold or more [15].

Sleep disorders are common in diabetic patients, increasing their morbidity and mortality [34]. Disturbed sleep alters insulin sensitivity, leading to hyperglycemia [35]. In response to poor sleep, the hypothalamic-pituitary-adrenal system increases glucocorticoid production, leading to increased glucose production and reduced glucose consumption, worsening glycemic control [36]. In addition, polyuria and nocturia, which are characteristic of diabetes, are major causes of sleep disorders, affecting sleep onset, and sleep maintenance, and aggravating these disorders [23]. As a result, diabetic patients often suffer from poor sleep quality due to physiological imbalances and associated sleep pathologies [23].

We hypothesize that sleep quality, in addition to being influenced by sociodemographic factors such as gender, age, and occupation, is also modulated by lifestyle habits (smoking, coffee consumption, and physical activity), disease-related characteristics (years of diabetes, complications, and treatment methods), emotional factors (depressive symptoms, anxiety, stress, fatigue, and concentration) and clinical parameters (BMI, HbA1c, fasting plasma glucose, and blood lipid profile). The nocturnal complications of diabetes have a direct impact on sleep quality, and sleep management is crucial for improving patients’ health and well-being. The present study aims to evaluate the effect of sleep quality on glycemic control and psychological factors in Moroccan adult type 2 diabetic patients and to explore correlations between subjective sleep quality, glycemic control, and psychological factors.

The present research revealed that 84.2% of the people surveyed had poor sleep quality, with a PSQI score > 5. Poor sleep quality was statistically greater in women than in men: 57.5% versus 26.7%, respectively. Earlier studies reported that women had poorer sleep quality than men did [37,38]. Older age and menopause may be linked to this phenomenon [39], babies’ crying, partners’ snoring, and daytime causes such as sleepiness or lack of concentration [40]. Notably, the women included in the study were younger than the men: 64.3 ± 9.60 years versus 67.7 ± 8.38 years, respectively.

The high incidence of poor sleep quality according to the PSQI score is consistent with the results of a study of diabetic patients in Jordan, where the PSQI score (PSQI>8) of patients was 81.0% [33]. Furthermore, the findings of several other studies are consistent with the results of the present study on poor sleep quality. In, Kenya (PSQI>5) (53.4%) [41], Singapore (47.1%) [23], Ethiopia (PSQI>5) (47.2%) [25], Iran (PSQI>5) (50.7%) [42], Qatar (PSQI>5) (35,8%) [43], Saudi Arabia (PSQI>5) (41,1%) [44], Brazil (PSQI > 5) (65,5%) [45] and Korea (PSQI>5) (49%) [46]. In addition, Cappuccio et al. reported that 47.1% of T2D patients were poor sleepers [47].

T2D patients have more sleep disturbances compared with controls (Lecube et al. 2016). Comparing studies using the PSQI index is complex due to differences in patient characteristics, such as age, diet, lifestyle, and sleep measurement thresholds. The complications of diabetes, such as neuropathy and nocturia, aggravate poor sleep quality [33]. In addition, sleep quality influences glycemic control, with established links between inadequate sleep duration and elevated glucose and glycated hemoglobin levels [48].

A person's sleep time could be crucial in determining the risk of developing metabolic syndrome, including T2D [49–51]. In the present study, the average sleep duration per day was 7 hours, 4 hours at 9.2%, 3 hours at 0.8%, 6 hours at 16.7%, and 5 hours at 6.7% suggesting that a majority of type 2 diabetics suffer from sleep disorders. In addition, our results show that 40.5% of patients declare having sleep disorder triggers of psychological origin (e.g., posttraumatic stress, emotional shock, mood disorders), physiological origin (e.g., illness, sleep apnea), while 16.5% of our patients define their sleep disorder triggers of environmental origin (e.g., stress, noise, bad bed), and the remaining 2.5% define them of hygienic origin (e.g., work schedule, medication, diet...). In this sense, 10.8% of diabetic patients reported using sleeping pills 3–4 times a week, and all of them were poor sleepers. However, only 0.8% reported taking sleeping pills at least once a week, which can alter the sleep-wake cycle and lead to stress [52].

In the current study, glycemic control, which was based on an HbA1c level of <7%, was reported in only 33.3% of the T2D patients. Similarly, other studies have reported that 24.3%, 39.1%, and 12.2% [44,46,49] of the patients with T2D expressed glycemic control. This is an indication of a major failure in the effective management of diabetes, even in a specialist hospital, which needs further investigation into the underlying causes [46,49].

Our findings revealed no significant correlation between HbA1c and sleep quality (OR = 1.13, P = 0.486), which is in line with several studies [8,53–55]. However, Cappuccio et al, reported a significant difference between good and poor glycemic control in relation to sleep quality [47] using PSQI. Other research has shown positive associations between HbA1c and sleep quality [33,56,57].

Tsai et al. revealed an inverse correlation between HbA1c and sleep quality, suggesting that improved glycemic control is linked to better sleep quality [46]. This relationship is probably due to the complications of diabetes [46], such as neuropathy and osmotic symptoms, which disrupt sleep by frequent visits to the toilet during the night [58]. Controlling blood sugar levels can be an effective therapeutic strategy for diabetic patients. To this end, it can be beneficial to set an appropriate sleep time to optimize patients’ sleep duration and help them improve the quality of their sleep.

Regarding the treatment type of diabetic patients, this study showed that 11.8% of diabetic patients on insulin complained of poor sleep quality, compared with 57.1% of oral hypoglycemic Agents (OHA) patients. This result contradicts the study by Cappuccio et al, who reported that insulin users more often complained of poor sleep quality than those on OHA alone did [47]. This finding is similar to the findings of Rajendran et al, who also reported no significant relationship between sleep quality and the type of diabetes treatment [59]. This may be explained by social and environmental factors, such as the average duration of diabetes, in addition to neuropathy and nocturia, which are more common in long-term diabetic patients. We suggest that the choice of antidiabetic medication in type 2 diabetic patients does not have a significant impact on sleep quality, regardless of other variables.

The role of sleep is to modulate glucose metabolism and energy absorption, which has a direct or indirect action on the maintenance of satisfactory glycemic control in patients with T2D. In addition, the increased prevalence of low levels of glycemic control in cases of sleep deprivation and/or poor-quality sleep could be due to upregulation of appetite, increased time spent eating, and reduced energy expenditure. In addition, nocturnal arousal and excitement could be associated with altered leptin levels and leptin resistance, leading to dysregulation of the hypothalamic-pituitary-adrenal (HPA) axis and resulting in altered glucose metabolism [15].

The two best-known pathways by which sympathetic nervous system activation is likely to predispose individuals to obesity, altered blood glucose levels, metabolic syndrome, and T2D are the inhibition of leptin secretion and stimulation of the HPA axis, leading to excessive cortisol secretion, which in turn alters glucose homeostasis [15].

The pathophysiological pathways linking sleep to diabetes mellitus are not simple linear cascades but complex cascades that involve neuroendocrine and metabolic networks [15]. The sympathetic nervous system and the HPA axis are activated during sleep disorders, resulting in increased cortisol secretion. In addition, this glucocorticoid has adverse effects on metabolism, such as insulin resistance and metabolic syndrome, by increasing glucose production and decreasing its utilization. Thus, sleep disorders can alter carbohydrate metabolism, favoring the risk of T2D, and can be caused by hyperactivity of the HPA axis, leading to various sleep disorders [15].

In our patients, comorbidities such as arterial hypertension (67.5%), dyslipidemia (40%), obesity (45.8%), hyperuricemia (45%), autoimmune diseases (11.7%), and cardiovascular diseases (33.3%) can lead to poorer sleep quality than the absence of comorbidities. However, comorbidities can lead to poor glycemic control, chronic complications, and increased emotional distress, which can result in disturbed homeostasis and poor sleep quality. While we did not find a significant association between BMI and sleep quality, this type of association was not materialized by significant rates after the application of logistic regression. This differs, however, from some previous studies that reported significant associations [47].

The results also showed numerical differences in the mean levels of fasting blood glucose (FBS), TC, and TG between patients with poor and good sleep quality; however, these differences were not statistically significant. Similarly, a study conducted by Khorasani et al. found that serum TG levels were higher in patients with poor sleep quality than in those with adequate sleep [60]. Based on experimental and clinical data, certain mechanisms have been suggested to link the relationship between sleep disorders and lipid metabolism. A decrease in leptin, a decrease in insulin sensitivity, an increase in sympathetic nervous system activation, and an increase in cortisol production have all been proposed as explanations [61,62].

Our data indicate that greater lipid disturbance is associated with higher levels of TC in poor sleepers, as observed by [63]. In addition, a significant correlation between sleep quality, as measured by the PSQI index, and HDL levels was found, which is consistent with the findings of [64], but differs from the study by Jennings et al., who did not observe this association [65].

HDL plays a key role in reverse cholesterol transport and possesses antioxidant and anti-inflammatory properties (Fanni et al. 2020). The lack of such relationships could be due to variations in sleep quality measurements across different studies. However, hyperglycemia and insulin resistance alter HDL particles, rendering them dysfunctional, as is frequently observed in T2D [66].

Furthermore, no association was found in our study between poor sleep quality in diabetic patients with no occupational status and poor sleep quality in employees. However, joblessness can lead to psychological disorders such as anxiety, low self-esteem, and depression. These consequences can translate into poor sleep quality and quantity [67,68]. This explains the high rate of poor-quality sleep in the unemployed group in this study (42.5%) compared with the non-employed group with good sleep quality (12.5%).

Our findings regarding the high prevalence of poor sleep quality and its relationship with anxiety are consistent with growing evidence suggesting that sleep and mental health may be influenced by broader metabolic and lifestyle factors. In this context, Shirased et al. reported that greater adherence to Mediterranean and DASH dietary patterns was associated with better sleep quality among women with overweight or obesity, while adherence to the Mediterranean diet was also associated with lower level of anxiety [69].

The present study showed that anxiety was a significant predictor of sleep quality, suggesting that for each one-unit increase in the GAD-7 anxiety score, the risk of poor sleep quality was multiplied by 1.21. This study, one of the first in the Moroccan context, also showed that anxiety and poor sleep quality negatively influence glycemic control, reinforcing the idea that clinicians should monitor these two variables in patients with T2D.

The results of the study indicated that there was a significant difference (P = 0.007) in GAD-7-rated generalized anxiety state when comparing groups of patients with good and poor sleep quality, and this variable was a significant major predictor (P = 0.005) of poor sleep quality in our T2D patients. The associations between poor sleep quality and anxiety were stronger than the associations between poor sleep quality and T2D and sleep quality and high blood pressure, respectively. Dong et al., also found a significant interaction between poor sleep quality and anxiety symptoms (GAD-7 ≥ 10) [69,70].

Inadequate sleep quality and anxiety both activate the HPA axis, stimulating the sympathetic nervous system and adrenal responses, increasing cortisol levels and inflammation, decreasing insulin sensitivity, and worsening glycemic control [69,70]. Both factors are also directly linked to impaired glycemic control [46], sleep quality and poor diabetes self-management, increased food intake, changes in time and quantity of food intake, a preference for energy-rich foods, impaired glucose tolerance, and insulin sensitivity [71]. In contrast, a chronic high-fat, high-protein diet and delicious foods can raise fasting blood glucose levels, which can intensify anxiety [72]. Anxiety disorders are also associated with poor diet adherence, inadequate glycemic control, and increased adrenergic activity [73]. These findings suggest that the combination of poor sleep quality and anxiety may affect quality of life in people with T2D [70].

It's also worth noting that recent work has highlighted the bidirectionality between sleep disorders and anxiety, with anxiety being predictive of sleep disorders and vice versa [74]. In this regard, findings suggest that clinicians investigating anxiety complaints in T2D patients should investigate poor sleep quality, and vice versa. Behavioral and pharmacological treatments are effective for anxiety disorders and poor sleep quality in people with T2D [75,76].

Furthermore, a recently published meta-analysis established the association between diabetes and an increased risk of cognitive impairment and dementia [77]. The reason for this is that inadequate control of diabetes, along with increased plasma glucose concentrations, can lead to cognitive impairment. Not only is hyperglycemia dangerous because of the long-term complications it can cause, including cardiovascular diseases, but it also has a direct negative impact on the central nervous system (CNS), contributing, among other things, to the slowing down of cognitive functions, and can also be associated with sleep disorders [78]. In this regard further investigation needed to explore the association of cognitive dysfunction and anxiety in diabetic patients with poor sleep quality.

## 5. Conclusion

This study demonstrates that the total prevalence of poor sleep quality in diabetic patients was 84.2%, with the mean ±SD of the global PSQI score (9.18 ± 3.73), highlighting the importance of assessing sleep quality in patients with T2D. Regarding GAD-7 score, 43.3% of participants were classified as anxious with the mean ±SD of the global GAD-7 score (8.0 ± 6.11). In addition, our findings demonstrate that’s 66.66% of the participants have an uncontrolled HbA1c (≥7), with Mean±SD (8.02 ± 1.63). Our results revealed a highly significant association between poor sleep quality and high levels of anxiety, as measured by the GAD-7 scale in the diabetic patients, independent of their glycemic control according to the Multivariate model used. These findings underline the crucial role of psychological factors in diabetes management. In conclusion, it is essential to integrate sleep and anxiety assessments into the care of diabetic patients to improve their overall well-being and optimize their treatment.

### Limitations

Firstly, the present study was based on a group of patients with T2D and without a control group, whereas studies like a case-control study and an equal percentage gender participants can boost statistical power. Secondly, in this study, it is difficult to examine the causal relationship because both variables are examined simultaneously and it is difficult to know which appeared before the other. For this reason, we will be studying this bidirectional relationship in an experimental animal model in future research. No objective tool was used to measure sleep quality, and sleep duration was estimated using a self-reported questionnaire. we found no significant correlation between HbA1c or BMI and sleep quality, which may be attributed to the small sample size of our study. Finally, recruiting participants from a single military hospital, even so, it may be representative, given that it is home to most of Morocco's specialists, may limit the representativeness of the sample and, consequently, the generalizability of the results to the entire Moroccan population with T2D.

### Recommendations

Based on the findings of the current study, the following specific recommendations are made: healthcare professionals should pay greater attention to early detection of poor sleep quality in patients with type 2 diabetes and consider appropriate measures to improve sleep quality when necessary. A more in-depth assessment of sleep quality in type 2 diabetic patients using objective methods such as polysomnography in addition to the subjective tool to obtain more reliable results. Providing additional intervention for type 2 diabetic patients with poor sleep quality. More stringent interventions should be offered to for patients with poor glycemic control. To gain a clearer idea of the situation, a full survey including type 2 diabetic patients from other healthcare organizations in Morocco is required.

## Supporting information

S1 File Graphical abstract. (DOCX)

## Abbreviations

BMI: Body Mass Index
CNS: Central Nervous System
DM: Diabetes Mellitus
FBG: Fasting Blood Glucose
GAD: Generalized Anxiety Disorder
HbA1c: glycosylated hemoglobin A1c
HDL: High-Density Lipoprotein
HMIMV: Mohamed V Military Instruction Hospital
HPA axis: Hypothalamic-pituitary-adrenal axis
ICSD: International Classification of Sleep Disorders
IDF: International Diabetes Federation
LDL: Low-Density Lipoprotein
OHA: Oral hypoglycemic Agents
OR: Odds Ratio
PSQI: Pittsburgh Sleep Quality Index
Rho: Spearman's Correlation Coefficient
SNS: Sympathetic Nervous System
T2D: Type 2 Diabetes
TC: Total Cholesterol
TG: Triglycerides
WHO: World Health Organization
