# Supplementary material for: Poor sleep quality and anxiety in patients with diabetes mellitus and its association with glycemic control: A cross-sectional study
Source: PLoS One. 2026 Nov 11;21(11):e0361467. doi: 10.1371/journal.pone.0361467 (PMC13754237; doi:10.1371/journal.pone.0361467)
Supplement: Supplementary file 1 [file pone.0361467.s001.docx]

**Graphical Abstract**

***Created by: WISSAL BAGHDAD***
